# What is the hologenome concept of evolution?

**DOI:** 10.12688/f1000research.14385.1

**Published:** 2018-10-19

**Authors:** J. Jeffrey Morris

**Affiliations:** 1 Department of Biology, University of Alabama at Birmingham, Birmingham, Alabama, USA

**Keywords:** microbiome, holobiont, symbiosis, hologenome

## Abstract

All multicellular organisms are colonized by microbes, but a gestalt study of the composition of microbiome communities and their influence on the ecology and evolution of their macroscopic hosts has only recently become possible. One approach to thinking about the topic is to view the host–microbiome ecosystem as a “holobiont”. Because natural selection acts on an organism’s realized phenotype, and the phenotype of a holobiont is the result of the integrated activities of both the host and all of its microbiome inhabitants, it is reasonable to think that evolution can act at the level of the holobiont and cause changes in the “hologenome”, or the collective genomic content of all the individual bionts within the holobiont. This relatively simple assertion has nevertheless been controversial within the microbiome community. Here, I provide a review of recent work on the hologenome concept of evolution. I attempt to provide a clear definition of the concept and its implications and to clarify common points of disagreement.

## Introduction

All multicellular life lives in symbiosis—in association, beneficial, detrimental, or neutral—with microbes. Indeed, the very first multicellular organism to evolve was already, in its genesis, colonized by microbes, and throughout the subsequent 3 or more billion years, the evolutionary landscapes of all multicellular species have been shaped by the microbes with which they share their environment. Since Koch and Pasteur popularized the germ theory of disease in the 19th century, the lay understanding of microscopic life has been primarily as a source of illness and decay ^1^. Secondarily, the special relationships between some types of plants and animals with mutualistic microbial symbionts—for example, corals and zooxanthellae ^2^, legumes and nitrogen-fixing bacteria ^3^, and squid and bioluminescent *Vibrio fischeri* ^4, 5^—have received ample attention. The relative ease of isolating many of these individual pathogens and mutualists into pure cultures is likely why their study dominated microbiology for most of its history.

However, since the first observations of microbes by Leeuwenhoek centuries before germ theory, it has been clear that microbes are enormously diverse and ubiquitously present, both living freely in the environment and occupying every possible space in and on animal bodies. Scientists have often speculated about the significance of our microbial fellow travelers, and since the late 19th century, the possibility that both normal and pathological microbiomes exist has loomed over the study of medical microbiology ^6^. However, the complexity of microbiome communities relative to single-species diseases (or mutualisms) has limited our ability to study the microbiome. Progress on this front did not pick up steam until the advent of high-throughput sequencing technologies in the 21st century. Now, we have the ability to rapidly and cheaply quantify the relative abundances of different microbial taxa in virtually any environment, and we are starting to develop an understanding of what kinds of microbes inhabit different macroscopic organisms. Microbiome communities range from well-defined, host-controlled populations of relatively low diversity (for example, pea aphids ^7^) to complex assemblages that, to some degree, persist through time (for example, human gut communities ^8^) to entirely transient, diet-controlled populations (for example, caterpillars ^9^).

How does the inevitable presence of the microbiome influence the evolution of both host and microbial residents? Theory suggests that intraspecies competition in a community context can favor the evolution of interspecies dependencies ^10^ and that mutualisms, once even weakly established, are fueled by positive frequency dependence, greatly increasing their chance of evolutionary fixation in a population ^11^. Classic examples of microbial symbioses clearly illustrate the stability of mutualisms once evolved, but, as with studies of human pathogens, they focus on only the most conspicuous interactions within a diverse, dynamic microbiome. What role, if any, do the other microbial taxa play in the ecology and evolution of the host?

One way of thinking about this question is to take a holistic view and see the host–microbiome system as a holobiont ^12^, or a compound organism consisting of the macroscopic host along with its all of its symbiotic microbes. An increasingly popular way of thinking about microbiome–host ecology and evolution is expressed by the hologenome concept, which maintains that the physiology of any macroscopic organism derives from the integrated activities of its own genome and all the genomes of its microbiome, that in many cases the microbiome is at least partially heritable, and that evolution can operate on the host or its microbiome (or both) because changes in either (or both) of them may impact the function of the holobiont ^12– 14^. Thus, the nuclear genome of an animal or plant comprises a relatively small fraction of the organism’s total genetic repertoire, and the majority are provided by microbes. These microbes can be obtained vertically (from the host’s parents) or horizontally (from the environment, other host species, other members of the same species, or kin in social species) and by influencing the phenotype of the holobiont they fundamentally alter the host’s evolution and ecology. Because macroscopic hosts are holobionts composed of many bionts and their respective genomes, the hologenome concept proposes that the “holobiont, the individual host organism, each of the diverse microorganisms, and the multitude of genes present in the hologenome” ^12^ are all potential targets of selection ^15^.

The hologenome concept was first applied to corals as it became clear that some diseases of these endangered keystone organisms resulted not from the invasion of specific pathogens but rather from the development of pathogenic microbiome communities, possibly caused by anthropogenic changes in their environment ^16^. Corals in particular were a fertile ground for hologenome thinking; on a microscopic level, the coral-zooxanthellae symbiosis was well known, but also the larger coral reef ecosystem was rife with mutualisms at all levels, encouraging the development of a “Russian doll” model of co-evolutionary feedbacks across different levels. As more work was done in the coral system, however, unique theoretical consequences of the hologenome concept began to come to light, and it became clear that the hologenome concept was applicable throughout the tree of life, potentially impacting the study of the ecology and evolution of every macroscopic organism on the planet ^17^. Attempts to apply the hologenome concept more broadly, however, have been controversial ^18, 19^. Here, I review the theoretical and empirical support for the hologenome concept and also attempt to give fair treatment to the various criticisms of the concept with an eye toward suggesting ways to resolve disagreements in the field.

## From concept to theory: progress in defining the holobiont

Today, adoption of some variety of the hologenome concept by microbial evolution and microbiome researchers is increasingly common. The words “hologenome” and “holobiont” are routine in the literature and have been applied to a great diversity of organisms, including corals ^20, 21^, insects ^22– 26^, sponges ^27^, cnidarians ^28, 29^, land plants ^30, 31^, seaweeds ^32^, macroscopic filamentous cyanobacteria ^33– 35^, humans ^36^, vampire bats ^37^, and even large unicellular protists ^38^. One large study provided evidence for the existence of host phylogenetic signals on microbiome composition in 31 animal species representing five different groups, including primates, and, in microbiome transplant experiments, interspecific microbiomes were deleterious to host performance and survival ^39^. Nevertheless, the concept has been controversial with some microbiome researchers ^18, 19^, and a lack of synthesis regarding the definition and demarcation of the holobiont and hologenome appears to persist. For instance, few would argue that the pea aphid and the bacterium *Buchnera aphidicola*, which live in a tight, vertically transmitted symbiosis with each other, are not linked evolutionarily. However, the definition of a holobiont is the “individual host and its [entire] microbial community” ^15^, including both obligate endosymbionts like *Buchnera* as well as all other facultative bacteria, archaea, protists, and viruses living in or on the organism. Therefore, the pea aphid holobiont will include many taxa that are only loosely affiliated with the host or indeed are entirely transient and may have little lasting influence on the host phenotype.

If the hologenome concept is to have any predictive value, the clear evolutionary difference between obligate symbionts like *Buchnera* and transient microbes must be quantifiable. One way this has been attempted is by relating the hologenome concept with familiar genomic evolution by equating changes in the frequencies of individual microbes in a holobiont with changes in the frequencies of nuclear alleles in the host population ^15^. This analogy implies that ecological shifts in microbiome community structure are mathematically similar to intergenerational changes in genomes ^40^ and can evolve because of either natural selection or random chance/drift. Under this “microbe = gene” paradigm, changes in the abundance of a specific taxon can be viewed as analogous to copy-number variation in a genome, and biont interactions in the holobiont can be similarly compared with genetic epistasis within the genome. Conspicuous symbionts are analogous to key regulatory genes—network hubs from a systems perspective. In contrast, organisms that are rarer, or present less often, are analogous to weakly expressed or inactive genes, generally evolving by drift but available for selection under an altered environment. The ultimate phenotype for the holobiont manifests as the product of all of these interspecific epistatic GxGxE effects, integrated across all members of the holobiont. A central focus of current hologenome thinking is to ask how strong or persistent these effects must be before selection can favor the evolution of specific host–microbe partnerships ^41, 42^.

**Figure 1.**
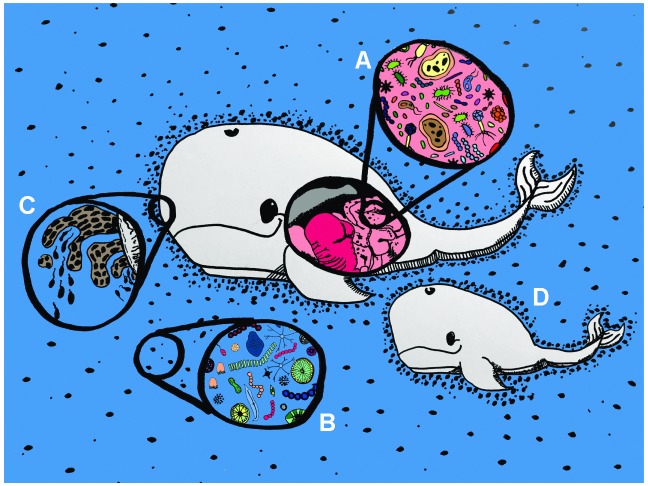
Challenges in defining the holobiont. Some of the discomfort over the hologenome concept stems from the difficulty in defining what a holobiont is. This mother and baby whale highlight some of the issues. ( **A**) According to the most common definition of “holobiont”, the microbes and viruses living inside the whale’s body clearly qualify as members of the whale holobiont. ( **B**) Likewise, at a sufficient distance from the whale’s body, the planktonic microbial population is clearly *not* part of the whale holobiont. ( **C**) However, many organisms spend part of their lives in contact with the whale and part of their lives elsewhere, as demonstrated by these skin microbes alternating between biofilm and planktonic life stages. The theoretical basis for understanding the evolutionary trajectories of these part-time symbionts remains in its infancy. ( **D**) The hologenome concept posits that many social animals inherit their microbiome from their kin because the environment they share becomes enriched with bacteria specific to their lineage (shown here as denser clouds of microbial dots near the whales). However, this presents interesting challenges to the hologenome concept since microbial symbiont lineages are shared more or less promiscuously between individual hosts and also spend time in the environment between hosts. In the case of these aquatic animals, the portion of the symbiont community currently living planktonically is even capable of communicating with those living on or in the whale via diffusible chemical cues (for example, quorum-sensing autoinducers). These factors blur the boundaries between holobionts and present difficulties for understanding evolution at the holobiont level by using traditional individual-based models. Artwork by Sarah J. Adkins.

A similar concern is that the physical line between the holobiont and the rest of the world is somewhat unclear: can the hologenome concept be expanded in scale to include larger ecosystems, like entire forest stands or oceans ( Figure 1)? Bordenstein and Theis address this question in the context of macroscopic mutualists, such as pollinators and flowers, and point out that these are clearly cases of separate holobionts interacting ^43^. However, other examples are less obvious. For instance, if the organisms in a forest stand, both plants and microbes, exchange metabolites, compete, or cooperate at a microscopic scale through the soil, could the entire stand be viewed as a single holobiont, with multiple hosts united through a partially shared microbiome? Must the microbiome be in direct, permanent contact with the host, or is there some spatial region near the host at which organisms still qualify as part of the microbiome? This is no mere semantic quibble, as it includes biogeochemically critical environments such as the rhizosphere in soils where chemical gradients created by the activity of roots harbor a distinct microbial community ^30, 44^ or the phycosphere ^45, 46^ near planktonic protistan algae where photosynthetic products support an enriched microbial ecosystem ^33– 35, 47^ and where selection for reduced sinking rates may favor microbiomes that linger close to hosts without directly attaching ^48^. Both of these environments are enriched with host-interacting microbes that have clear parallels with the internal microbiomes of animals (is there an important difference between being 1 cm from a root or 1 cm from the edge of the intestinal lining?), but the lack of a hard spatial boundary defining which microbial populations are host-associated and which are not creates new opportunities for understanding these important systems in a hologenomic context.

Some criticism of the hologenome concept appears to stem from conflation of this concept and other related multi-organism evolutionary concepts. Theis *et al*. ^15^ state that holobionts and hologenomes are not the same as “superorganisms” (collectives of multiple, often clonal, individuals of the same species, such as colonial ants) or “metagenomes” (the sum of all genetic information in an environment, irrespective of association with a host). Other critics appear to identify the hologenome concept with the literature on major evolutionary transitions in individuality, which focuses on the factors necessary to overcome conflict between formerly free-living partners to evolve a new multi-organism individual ^49– 52^. For instance, common critical statements suggest that the lack of evidence for reliable transmission of the intact microbiome ^18, 19^ or the lack of functional coordination or unified purpose within the holobiont ^53^ either invalidates the hologenome concept or renders it superfluous for the understanding of host evolution. Indeed, most microbiome members do not appear to be reliably vertically transmitted, preventing the kind of co-evolution that can lead to “egalitarian major transitions” ^49, 50^—the evolution of multiple disparate lineages into a single reproductively synchronized “organism”, as in the origin of mitochondria and chloroplasts, and certain holobionts like the pea aphid and *Buchnera*. Critics of the hologenome concept point to this fact as a central argument against the idea that the holobiont is a “unit of selection” ^19^. The originators of the hologenome concept acknowledge that the claim that “genomes of both hosts and a significant fraction of microbiomes are transferred between generations” is contentious ^14^, but they also maintain that neither functionally unified co-evolution nor faithful transmission of the entire microbiome is necessary for selection to operate at the holobiont level ^14, 43^. Theory demonstrates that natural selection can reinforce host–microbe associations that are propagated by mixed modes of vertical and horizontal transmission ^40^ and symbioses covering the entire spectrum between casually facultative and mutually obligate can be evolutionarily stable ^52, 54^. Under the hologenome concept, hosts whose microbiomes are not faithfully transmitted, by either maternal transmission or environmental acquisition, will see their microbiomes evolving like a genome experiencing genetic drift—randomly and without the ability to adapt. Indeed, if a host’s phenotype is strongly affected by its microbiome yet it cannot reliably propagate that microbiome to the next generation, then it is likely that its hologenome restricts its ability to adapt, much like widespread epistasis can impede evolution of quantitative traits because of the recombinatory scrambling of alleles in each generation. For this reason, there is possibly a strong evolutionary pressure for hosts to establish control over the structure of their microbiome—in effect, keeping its hologenome “on a leash” ^42^.

Critics sometimes suggest that the hologenome concept maintains that the holobiont is the most important or only level of selection ^18^, but according to proponents this is expressly not a condition of the hologenome concept. The notion that holobionts are units of selection does not preclude or minimize selection at the level of the biont, or the individual selfish gene, nor does it suggest that selection must proceed at the same pace or in the same “direction” for both host and microbiome. Recent theoretical advances in describing evolution at the holobiont level attempt to unite all of these disparate forces: interspecies competition within the microbiome, host incentives to control the microbiome through reliable vertical transmission of symbionts with positive effects on host fitness, and differential evolutionary rates for hosts versus microbiome members ^41, 42, 55^.

## Skepticism and future directions

In the above paragraphs, I have outlined the core elements of the hologenome concept and I have also tried to faithfully characterize the back-and-forth debate that has occurred over the past several years between proponents and critics of the concept. This debate has clearly led to refinement and clarification of the hologenome concept, but just as clearly there remains a substantial amount of dissatisfaction with the idea. In this section, I will consider three questions that consistently appear in criticisms of the hologenome concept.

### 1. Is the hologenome concept just a restatement of well-established ecological and evolutionary theory?

Some argue that the evolutionary dynamics described in the hologenome concept are merely special cases of well-established theories of co-evolution. For instance, is there any fundamental difference between the evolutionary dynamics linking host and microbial symbiont and those linking wolves and deer, cows and grass, or bacteria and bacteriophage? Do we gain anything new by taking a gestalt view of the many thousands of commonplace ecological interactions and evolutionary pressures inside a given host ecosystem? The answer to this question may be simply a matter of taste or focus—after all, it is the hologenome concept, not the hologenome theory, so it may not be a fatal flaw if its influence is primarily philosophical. Does the hologenome concept change the kinds of questions that researchers ask about the microbiome? If so, it may be useful even if it contributes no completely novel theory. That being said, supporters of the hologenome concept have begun to develop a mathematical framework for thinking about holobiont evolution ^41^, and a number of other theoretical papers have emerged that, while not specifically supporting the hologenome concept, can be thought of as broadly supportive of the notion that thinking of the holobiont as a whole is a theoretically sound and productive approach ^42, 56^.

### 2. Do disagreements about hologenome definitions encourage bad reasoning?

If we read the literature on the hologenome concept and holobionts more broadly, it is clear that the definitions and significance of the terms “holobiont” and “hologenome” are understood differently by different authors. The very precise definitions recently outlined by Bordenstein *et al*. ^15, 43^ are nuanced, open to falsification, and admit that the microbiome (like all quantitative traits) is only partially heritable and subject to drift. However, a concern that is sometimes raised is that researchers in disparate fields who are not as comfortable with the game theoretical arguments working against interspecies cooperation within a holobiont may be misled by the hologenome concept into believing in a unified “superorganism” that does not exist. In their introductory paragraph, Douglas and Werren ^19^ cite several recent papers that they believe have misapplied the hologenome concept to draw erroneous conclusions about important topics (for example, honeybee decline and cancer biology). Indeed, the term “holobiont” has surfaced in a resurrection of the fanciful Gaia hypothesis ^57^—itself another concept put forward by Lynn Margulis, who coined the term “holobiont” ^58^. Although new conceptual frameworks might help stimulate new advances as mentioned in the previous paragraph, they can also lead to bad reasoning if they oversimplify or minimize important problems, especially if the new concepts become popular outside of their major field. Supporters of the hologenome concept should strive to “call out” poor applications of the concept in the scientific literature as well as the popular press and to ensure that publications about the concept deal openly with the “gray areas” of host–microbiome association instead of focusing on conspicuous cases of well-established symbioses (for example, corals).

### 3. The hologenome is a potential level of selection, but is it ever an important one?

This is perhaps the most common criticism of the hologenome concept and indeed of multilevel selection theory in general. To quote Douglas and Werren ^19^, “We do not argue that selection cannot act on the host-microbiome as a unit. We simply argue that evidence for this is weak, and the conditions necessary for it to occur are unlikely”. Indeed, if one understands “holobiont” to apply only to associations that approach “organismality”, with faithful vertical transmission and widespread cooperation, then this is undeniably true. Yet it is certain that these conditions have occurred numerous times in the history of life and are occurring right now in many well-studied symbioses; there must exist a path, and intermediate stages, leading to obligate symbiosis. Elsewhere, my colleagues and I have argued ^52^ that most microorganisms exist along a continuum between free-living and obligately symbiotic and that different evolutionary pressures favor movement toward one or the other extreme. Other theoretical treatments suggest that evolution favors host adaptations that allow control of the microbiome ^42, 55^, a process that can be seen as supportive of the hologenome concept. A central challenge for the hologenome concept will be to effectively understand this continuum and how (or whether) it influences the ability of selection to work on the level of the holobiont (for example, 41).

## Practical consequences of hologenomic thinking

What exactly is riding on whether or not the hologenome concept gains widespread acceptance? Perhaps the biggest concern is not that the hologenome concept has no merit but rather that it possibly exaggerates the importance of selection at the holobiont level to the evolutionary outcomes of the individual species. This may be interpreted as the most recent salvo in the “group selection” battles that have been fought since at least the 1970s, but from another perspective the resurrection of Lamarckian ideas (that is, acquisition of symbionts from the environment that become vertically inherited or evolution of microbes within a single host generation in response to environmental change) casts a shadow over many “settled” issues in evolutionary biology. As an example, much of our thinking about animal behavior arises from a Darwinian understanding of sexual selection, inclusive fitness, and life history evolution, yet a growing body of evidence suggests that many animal behaviors—including complex human behavioral syndromes like depression and autism spectrum disorder—are strongly influenced by the microbiome ^59^. Whether these behaviors arise because of co-evolved mutualisms between animals and particular microbes or because of opportunistic infections by microbes that manipulate our behavior for their own benefit or as a by-product of unrelated metabolisms, a proper understanding of the forces that control our relationship with our microbial fellow travelers will be a critical component of a modern understanding of what it means to be human ^60^. Indeed, perhaps the greatest contribution of the hologenome concept would be to raise the profile of the inner ecology of animals and plants and to bring into sharper focus the fusion of ecology and evolution that happens at this microscopic scale.

Understanding the degree to which animals and their microbiomes are interconnected evolutionarily is also relevant to human health. Since the 19th century, researchers have speculated that some illnesses result from pathological microbiome composition rather than infection by particular agents and also that susceptibility to many individual pathogens may be exacerbated by perturbed microbiomes ^6, 61^. Deviations from a “healthy” or “normal” microbiome also seem to be related to the ability of mosquitoes to transmit disease to humans ^24^, and changes in the metabolic state of microbiome members have even been shown to affect longevity in the roundworm *Caenorhabditis elegans* ^62^. These observations suggest that intentional manipulation of the microbiome (that is, microbiome engineering ^63^) could produce a wide variety of positive health outcomes for humans, but determining the most effective interventions could depend on the evolutionary relationship between host and symbionts. If host and microbiome are connected evolutionarily, then it is reasonable to expect that dysbiosis may be countered by removing whatever environmental stimulus or pathogenic agent has perturbed the “normal” community. On the other hand, if a host is a blank slate occupied by opportunistic microbial communities, then transitioning a patient to a healthy microbiome state may require re-inoculation with particular “good microbes” in addition to the removal of the “bad” ones. In the latter case, it may be most efficient for humans to occupy environments that are intentionally constructed so as to nurture “good” microbes, such that we are constantly re-infected by these beneficial microorganisms ^64^.

The hologenome concept of evolution is an intellectually satisfying effort to help evolutionary theorists make sense of the deluge of microbial ecology data that are becoming available in the era of high-throughput sequencing. Critics of the hologenome concept believe that this concept “presupposes” that the microbe–host system is selected as a unit, whereas, in their view, greater insight is gained by investigating how selection acts on the different members of the community. However, the volume of criticism directed against it is not necessarily evidence that the hologenome concept is unhelpful. Rather, the hologenome concept has combined elements from a wealth of theoretically contentious fields—multilevel selection theory, microbial systematics, the evolution of complexity, and social evolution—to produce a way of looking at life which is simultaneously exciting, confusing, and challenging. By providing a framework for thinking about the relationship of host and microbiome, it drives the generation of testable hypotheses in a field that has often seemed overwhelmed with strictly observational science. The degree to which the holobiont is an important unit for understanding evolution remains to be determined, but it is clear that the research driven by this concept will underlie many advances in the coming years.
